# Validation of an Overnight Wireless High-Resolution Oximeter plus Cloud-Based Algorithm for the Diagnosis of Obstructive Sleep Apnea

**DOI:** 10.6061/clinics/2020/e2414

**Published:** 2020-11-10

**Authors:** George do Lago Pinheiro, Andrea Fonseca Cruz, Diego Munduruca Domingues, Pedro Rodrigues Genta, Luciano F. Drager, Patrick J. Strollo, Geraldo Lorenzi-Filho

**Affiliations:** I Laboratorio do Sono, Divisao de Pneumologia, Instituto do Coraçao (InCor), Hospital das Clinicas (HCFMUSP), Faculdade de Medicina, Universidade de Sao Paulo, Sao Paulo, SP, BR; II Biologix Systems, Sao Paulo, SP, BR; III Unidade de Hipertensao, Instituto do Coracao (InCor), Hospital das Clinicas (HCFMUSP), Faculdade de Medicina, Universidade de Sao Paulo, Sao Paulo, SP, BR; IV Unidade de Hipertensao, Divisao Renal, Hospital das Clinicas (HCFMUSP), Faculdade de Medicina, Universidade de Sao Paulo, Sao Paulo, SP, BR; V Division of Pulmonary, Allergy and Critical Care Medicine, University of Pittsburgh, Pittsburgh, PA, USA

**Keywords:** Diagnosis, Obstructive Sleep Apnea, Oximetry

## Abstract

**OBJECTIVES:**

Obstructive sleep apnea (OSA) is a common but largely underdiagnosed condition. This study aimed to test the hypothesis that the oxygen desaturation index (ODI) obtained using a wireless high-resolution oximeter with a built-in accelerometer linked to a smartphone with automated cloud analysis, Overnight Digital Monitoring (ODM), is a reliable method for the diagnosis of OSA.

**METHODS:**

Consecutive patients referred to the sleep laboratory with suspected OSA underwent in-laboratory polysomnography (PSG) and simultaneous ODM. The PSG apnea-hypopnea index (AHI) was analyzed using the criteria recommended and accepted by the American Academy of Sleep Medicine (AASM) for the definition of hypopnea: arousal or ≥3% O_2_ desaturation (PSG-AHI_3%_) and ≥4% O_2_ desaturation (PSG-AHI_4%_), respectively. The results of PSG and ODM were compared by drawing parallels between the PSG-AHI_3%_ and PSG-AHI_4%_ with ODM-ODI_3%_ and ODM-ODI_4%_, respectively. Bland-Altman plots, intraclass correlation, receiver operating characteristics (ROC) and area under the curve (AUC) analyses were conducted for statistical evaluation. ClinicalTrial.gov: NCT03526133.

**RESULTS:**

This study included 304 participants (men: 55%; age: 55±14 years; body mass index: 30.9±5.7 kg/m^2^; PSG-AHI_3%_: 35.3±30.1/h, ODM-ODI_3%_: 30.3±25.9/h). The variability in the AASM scoring bias (PSG-AHI_3%_ *vs* PSG-AHI_4%_) was significantly higher than that for PSG-AHI_3%_ *vs* ODM-ODI_3%_ (3%) and PSG-AHI_4%_ *vs* ODM-ODI_4%_ (4%) (9.7, 5.0, and 2.9/h, respectively; *p*<0.001). The limits of agreement (2±SD, derived from the Bland-Altman plot) of AASM scoring variability were also within the same range for (PSG *vs* ODM) 3% and 4% variability: 18.9, 21.6, and 16.5/h, respectively. The intraclass correlation/AUC for AASM scoring variability and PSG *vs* ODM 3% or 4% variability were also within the same range (0.944/0.977 and 0.953/0.955 or 0.971/0.964, respectively).

**CONCLUSION:**

Our results showed that ODM is a simple and accurate method for the diagnosis of OSA.

## INTRODUCTION

Obstructive Sleep Apnea (OSA) is characterized by repetitive episodes of complete (apnea) or partial (hypopnea) upper airway obstruction, resulting in intermittent oxygen desaturation and fragmented sleep (1). OSA is associated with various symptoms such as habitual snoring, poor, and non-restorative sleep, excessive daytime sleepiness, and fatigue that ultimately have a negative impact on the quality of life (1). Untreated OSA is independently associated with cardiovascular diseases, such as hypertension, arrhythmias, progression of atherosclerosis, coronary artery disease, stroke, and cardiovascular death (2). OSA is extremely common in the general population, with a prevalence ranging from 9.6% in women to 49.7% in men, depending on the population characteristics (3-6). Polysomnography (PSG) is considered to be the gold standard for the diagnosis of OSA. However, PSG has limitations, because it is expensive, presents an inconvenience to patients, and may not be readily available in certain locations (7,8). Long waiting list also affect access to OSA diagnosis. The difficulties associated with diagnosis certainly contribute to the observation that the vast majority of patients with OSA in the general population are not diagnosed and therefore remain untreated (9). The estimated cost of the lack of recognition and treatment of OSA is approximately 150 billion dollars per year in the USA, due to the increased number of accidents, decreased productivity, and associated co-morbid diseases (10).

The awareness of the high prevalence of OSA has led to the popularization of the home sleep test (HST). The HST records a limited number of respiratory signals and provides patients with a more comfortable and cost-effective testing option. The performance of the HST is comparable to that of PSG among patients with suspected OSA (11). The level of under diagnosed cases of OSA remains unacceptable, despite the increased implementation of HST for the diagnosis of OSA, intended to enhance accessibility. The oxygen desaturation index (ODI), developed in line with the diagnostic simplifications for OSA, which is determined by the isolation of the oximetry channel from the full PSG, has been reported to possess a high sensitivity and specificity for the detection of OSA in children and adults (12,13). However, a recent systematic review showed a large discrepancy in the sensitivities and specificities of the ODI obtained from pulse oximeters compared to the reference apnea-hypopnea index (AHI) (14). These observations explain why pulse oximetry is largely viewed as a screening tool (15). On the other hand, it is also clear that the performance of oximeters is variable. Therefore, new technology may enable better diagnostic performance for OSA.

This study was designed to validate a new device consisting of a high-resolution wireless oximeter with a built-in accelerometer linked to a smartphone application and automated cloud algorithm for the detection of oxygen desaturation, described herein as Overnight Digital Monitoring (ODM) (Biologix^TM^). Thus, consecutive patients referred to the sleep laboratory with suspected OSA and no significant comorbidities underwent PSG and ODM on the same night. We reasoned that the variability in the AHI derived from the recommended *versus (vs)* accepted American Academy of Sleep Medicine (AASM) criteria for hypopnea sets the metric of permissible clinical variability, to validate ODM as a diagnostic tool for OSA. Hypopnea is defined as a 30% fall in airflow for at least 10 s. The recommended AASM criteria stipulate that airflow reduction should be associated with arousal or oxygen desaturation of at least 3%. Alternatively, AASM also accepts that hypopnea can be defined by airflow reduction associated with an oxygen desaturation of at least 4% (16). Therefore, we hypothesized that ODM was an accurate diagnostic modality for moderate-to-severe OSA and that the variability between the recommended and accepted AASM PSG-AHI criteria for hypopnea would not differ from the variability between the PSG-AHI and ODM-ODI.

## MATERIAL AND METHODS

### Patients

We assessed consecutive adult patients with suspected OSA, who were referred for full PSG at the sleep laboratory of the Heart Institute between July 2017 and July 2018 for eligibility for inclusion in this study. We excluded patients with diagnoses of heart failure, unstable clinical condition, decompensated chronic obstructive pulmonary disease, renal failure, hepatic disease, those on supplemental oxygen, those on continuous positive airway pressure (CPAP) titration, or patients participating in other studies. We also excluded patients who had less than 4 h of sleep. The local ethics committee approved the study protocol (SDC 4515/17/015) and informed consent was obtained from each participant.

### Sleep Studies

All patients underwent overnight in-laboratory PSG using the standard montage that included recording of the electroencephalogram (EEG) central (C) and occipital (O) channels referred to the auricular channel (A) (C3/A2, C4/A1, O1/A2, O2/A1), electrooculogram (EOG), submental electromyogram (EMG), left and right anterior tibialis EMG, electrocardiogram, thoraco-abdominal effort, oronasal airflow (thermistor and nasal pressure based airflow measurement), oxygen saturation (SpO_2_) with pulse oximetry, and body position (EMBLA S7000, Embla Systems, USA and Alice 5, Respironics Inc., USA). Participants wore a wireless oximeter (Oxistar™, Biologix Sistemas Ltd., Brazil) with a built-in accelerometer on another finger of the same hand as the PSG oximeter. The Oxistar™ firmware acquires 100 samples per second generating beat-to-beat raw data of SpO_2_ with a resolution of 0.1%. A moving time average of 4 cardiac beats were used. The data obtained from Oxistar™ were transferred via the smartphone application to the cloud, where the data were automatically analyzed using a proprietary algorithm. The results were expressed as the number of oxygen desaturations per recording hour. All PSG studies were scored by two independent certified technicians who were blinded to the ODM results. Hypopnea was defined as the peak signal excursion drop ≥30% of the pre-event baseline nasal pressure signal lasting ≥10 s. Respiratory events were scored independently by two technicians according to the recommended (≥3% reduction in SpO_2_ from the pre-event baseline or an event associated with arousal) and the acceptable AASM criteria for hypopnea (≥4% reduction in SpO_2_). The PSG-AHI criteria recommended and accepted by the AASM are described herein as PSG-AHI_3%_ and PSG-AHI_4%_, respectively (17). Mild, moderate, and severe OSA were defined according to the current standards (5≤AHI<15; 15≤AHI<30; and AHI≥30 events/h, respectively) (18). The ODI obtained from ODM was expressed as the number of desaturations per valid recording time and automatically analyzed within the cloud. The results of the automated ODM analysis were matched using the 3% and 4% desaturation criteria (ODM-ODI_3%_ and ODM-ODI_4%_, respectively) to facilitate comparison with the PSG-AHI_3%_ and PSG-AHI_4%_.

### Statistical analysis

The sample size was calculated using the nomogram proposed by Malhotra et al. (19), which yielded a required sample size of 290 patients for the anticipated sensitivity and specificity of 0.9, absolute precision of 0.05 with a 95% confidence level, and estimated prevalence of moderate-to-severe OSA of 50%. Data were expressed as the mean±standard deviation or median (25-75% interquartile), wherever appropriate. Intraclass correlation coefficient (ICC) and Bland-Altman plot analyses were used to assess the agreement between PSG-AHI variability and ODM-ODI, using the 3% and 4% criteria. The bias (mean difference) between the recommended and acceptable AASM hypopnea criteria (PSG-AHI_3%_ *vs* PSG-AHI_4%_) was compared with the bias between PSG-AHI_3%_ *vs* ODM-ODI_3%_ and PSG-AHI_4%_ *vs* ODM-ODI_4%_ using the Wilcoxon test. Receiver operating characteristic (ROC) and area under the curve (AUC) analyses were conducted to determine and compare the overall agreement between the three pairs of comparisons (PSG-AHI_3%_ *vs* PSG-AHI-_4%_, PSG-AHI_3%_ *vs* ODM-ODI_3%_, and PSG-AHI_4%_ *vs* ODM-ODI_4%_). This method was also used to determine the best ODM cutoff for the diagnosis of moderate-to-severe OSA (AHI >15/h) by determining the sensitivity, specificity, positive predictive value (PPV), negative predictive value (NPV), positive likelihood ratio (LR+), negative likelihood ratio (LR-), and accuracy. Frequencies were compared using McNemar’s test. All statistical analyses were performed using the SPSS Statistics 24 software (IBM Corp., USA).

## RESULTS

**Figure 1:**
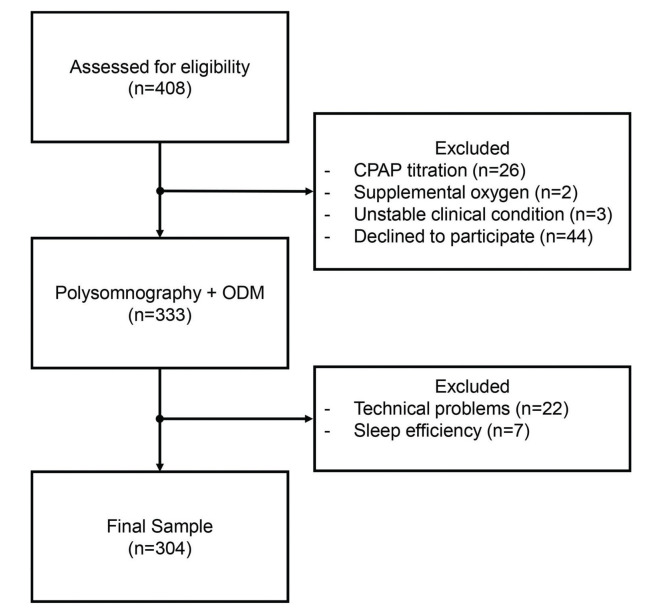
Flow diagram of the participant recruitment process. ODM=overnight digital monitoring.

**Figure 2:**
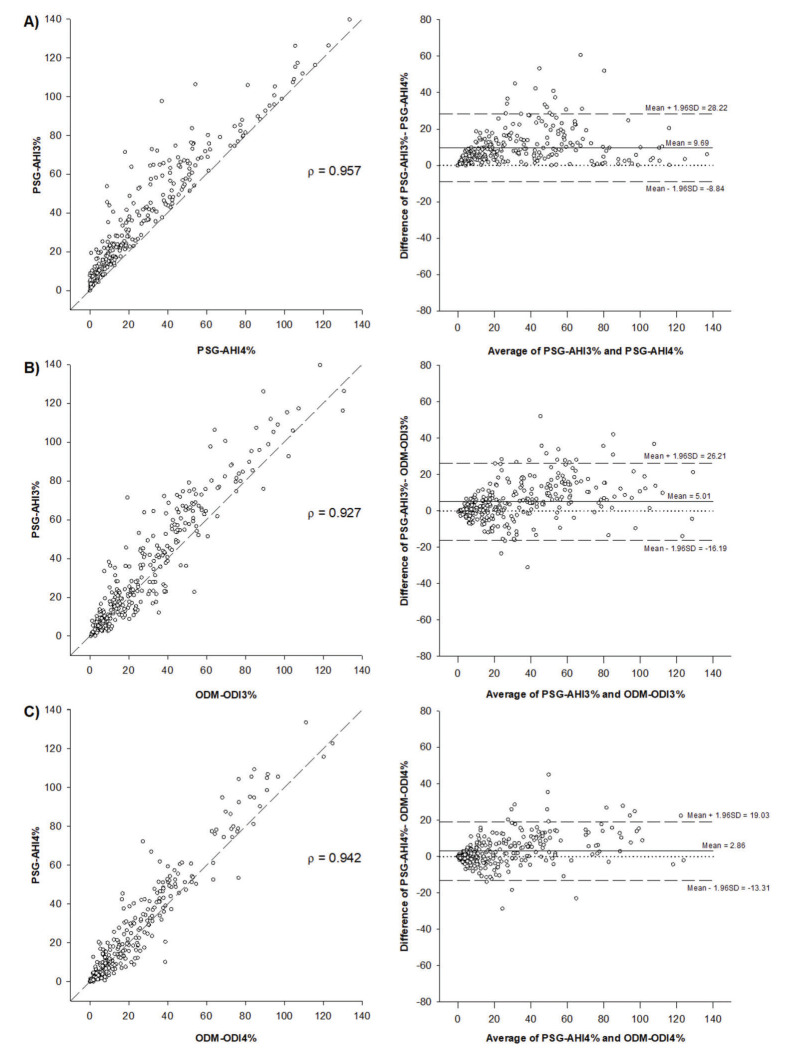
Scatter plots and Spearman’s correlation (r) and Bland-Altman plots comparing A) PSG-AHI3% and PSG-AHI4%, B) PSG-AHI3% and ODM-ODI3%, and C) PSG-AHI4% and ODM-ODI4%.PSG=polysomnography; AHI=apnea-hypopnea index; ODM=overnight digital monitoring; ODI=oxygen desaturation index.

**Figure 3:**
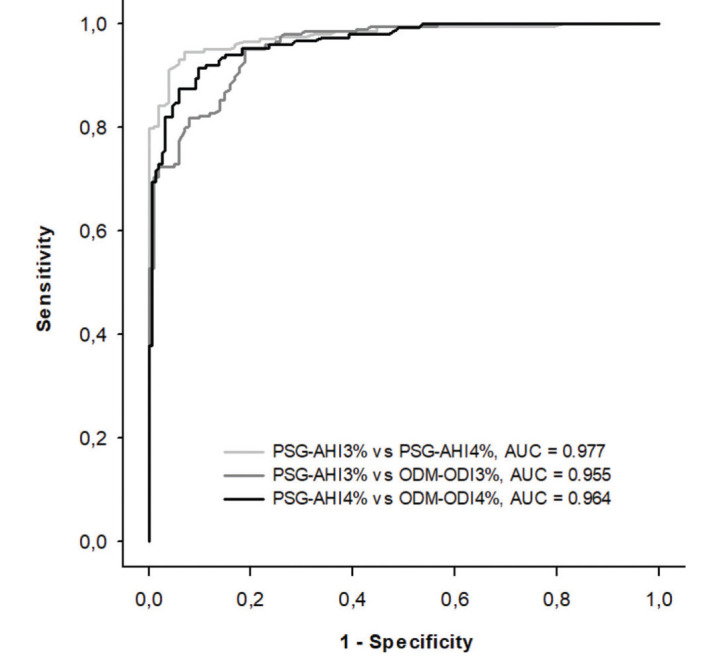
Receiver-operator characteristic curves of PSG-AHI3% vs PSG-AHI4% (light gray line), PSG-AHI3% vs ODM-ODI3% (medium gray line), and PSG-AHI4% vs ODM-ODI4% (black line) PSG=polysomnography; AHI=apnea-hypopnea index; ODM=overnight digital monitoring; ODI=oxygen desaturation index; vs=versus.

We screened 408 patients, and 104 were excluded for various reasons (Figure 1). The final sample consisted of 304 middle-aged patients (of both sexes) with obesity and frequent comorbidities (Table 1). The frequency of moderate-to-severe OSA determined by PSG reduced from 66.8% to 49.7% for the AASM recommended and accepted criteria (PSG-AHI_3%_ *vs* PSG-AHI_4%_) (*p*<0.0001). The bias (mean difference) between PSG-AHI_3%_ *vs* PSG-AHI_4%_ was significantly higher than that between PSG-AHI_3%_ *vs* ODM-ODI_3%_ and the bias between PSG-AHI_4%_ *vs* ODM-ODI_4%_ (9.7, 5.0 and 2.9, respectively; *p*<0.001). The limits of agreement (2±SD) created by the Bland-Altman plot for the comparison of PSG-AHI_3%_ and PSG-AHI_4%_ were within the same range as the limits of agreement between PSG-AHI_3%_ *vs* ODM-ODI_3%_ and PSG-AHI_4%_ *vs* ODM-ODI_4%_ (18.9, 21.6, and 16.5; Figure 2A, 2B, and 2C, respectively). The AUC for the diagnosis of moderate-to-severe OSA determined by PSG-AHI_3%_ *vs* PSG-AHI_4%_, PSG-AHI_3%_ *vs* ODM-ODI_3%_, and PSG-AHI_4%_ *vs* ODM-ODI_4%_ was 0.977, 0.955 and 0.964, respectively; Figure 3). The best cutoff for the detection of moderate-to-severe OSA using PSG-AHI_3%_ and PSG-AHI_4%_ (*i.e.*, the gold standard) was 12 and 14 events/h for ODM-ODI_3%_ and ODM-ODI_4%_, respectively. The frequency of the diagnosis of moderate-to-severe OSA using ODM-ODI_3%_ and ODM-ODI_4%_ was 62.2% and 51.3%, respectively, based on the best cutoff values. Tables 2A, 2B, and 2C present the four-class confusion matrix comparing the classification derived from the three pairs of parameters: PSG-AHI_3%_ *vs* PSG-AHI_4%_, PSG-AHI_3%_ *vs* ODM-ODI_3%_, and PSG-AHI_4%_ *vs* ODM-OD_4%_, respectively. The statistical metrics for the diagnosis of moderate-to-severe OSA demonstrated good performance for ODM-ODI_3%_ and ODM-ODI_4%_ (Table 3). The ICC for PSG-AHI_3%_ *vs* PSG-AHI_4%,_ PSG-AHI_3%_ *vs* ODM-ODI_3%_, and PSG-AHI_4%_ *vs* ODM-ODI_4%_ was 0.944, 0.953, and 0.971, respectively.

## DISCUSSION

Our study showed that a high-resolution wireless oximeter, linked to a smartphone and automated cloud algorithm for detection of desaturations, is a reliable method for OSA diagnosis and determination of OSA severity in patients with suspected OSA and determination of its severity among patients with suspected OSA. This conclusion is based on the good performance of ODM for the detection of moderate-to-severe OSA. Moreover, the variability of the main result derived from ODM compared to PSG (ODI and AHI, respectively) is at least similar to the clinical variability in the AHI permitted by the AASM arising from the use of the recommended or acceptable criteria for the definition of hypopnea (PSG-AHI_3%_ and PSG-AHI_4%_, respectively). First, we showed that the bias (mean difference) between PSG-AHI_3%_ *vs* PSG-AHI_4%_ was significantly higher than that between PSG-AHI_3%_ *vs* ODM-ODI_3%_ and PSG-AHI_4%_ *vs* ODM-ODI_4%_. Second, the variance (2±SD) between PSG-AHI and ODM-ODI was similar to the PSG variance derived from the different criteria for defining hypopnea (recommended *vs* acceptable) permitted by the AASM as shown by the Bland-Altman plots (Figure 2). Finally, the ROC curve, AUC, sensitivity, specificity, accuracy, PPV, NPV, LR+, and LR- of ODM were high for the diagnostic test designed to detect moderate-to-severe OSA (Table 3).

The concept that PSG is the gold standard for the diagnosis of OSA has been recently challenged (20). The traditional method for describing OSA using the metric of the number of apneas and hypopneas per hour of sleep (AHI) may not encompass all aspects of the OSA burden that may be better described, for instance, by the analysis of the oxygen signal (21,22). For instance, a recent study showed that the hypoxic burden is a major predictor of cardiovascular disease-related mortality, suggesting that clinical symptoms and oximetry data alone may play a major role in the management of patients with suspected OSA (21). Moreover, the recognition of respiratory events by PSG is highly dependent on the nasal cannula signal that provides a semi-quantitative measure of airflow. Therefore, it is not surprising that the definition of hypopnea is still being debated and not standardized, in contrast to that of apnea, which is unequivocal. The definition of hypopnea relies on more robust variables such as the presence of arousal or level of associated oxygen desaturation, owing to the limitation in the objective measurement of airflow. The AASM recommends that hypopnea must be defined whenever a 30% fall in airflow is associated with arousal or oxygen desaturation of at least 3% (16). The AASM also accepts a more stringent hypopnea definition that requires a 4% or greater decrease in oxygen saturation and ignores arousal (16). Although the choice of hypopnea definition has a substantial effect on the AHI and number of patients diagnosed with OSA, there is no standard adapted threshold value of AHI (23). Despite the recommended AASM criteria, several agencies, including the Centers for Medicare and Medicaid Services, continue to require a more stringent hypopnea definition, requiring a 4% or greater decrease in oxygen for the diagnosis of OSA. Oximetry provides a robust signal and tracks the pivotal consequences of respiratory events, which are actually the chief source of the variability in the hypopnea definition (3% *vs* 4% desaturation). The observation that the main result of ODM-ODI exhibited agreement with the PSG-derived AHI provides strong evidence of the reliability of ODM for the diagnosis of OSA among patients referred to the sleep laboratory due to a high probability of OSA.

In our study, the diagnostic performance of ODM for detecting moderate-to-severe OSA was similar to that of HST (24) (AUC=0.955 *vs* 0.891, respectively). HST is widely used and accepted for the diagnosis of OSA (25). ODM may be potentially beneficial for the large-scale diagnosis of OSA because it does not require special infrastructure, professional assistance for preparing patient for the test, and additional time for data analysis. We anticipate major reductions in the cost of the OSA diagnosis and the possibility of accessing under-served areas where PSG and HST are not readily available. The system operates over Wi-Fi or mobile networks. Mobile networks are widely available across large countries such as Brazil and India. The solution may also be helpful in First World countries. For instance, it is estimated that 23.4 million individuals with OSA (representing 80% of the American population with OSA) remain undiagnosed (11). Therefore, a simple diagnostic system for OSA may be helpful worldwide. A large randomized trial among patients with suspected OSA reported poorer outcomes when only oximetry was disclosed to the physicians (26). However, the authors acknowledged that poorer outcomes with oximetry data may be partially explained by lower physician confidence. Another important advantage of ODM over most HSTs is the fact that the sleep study can easily be accessed from the cloud and repeated as many times as necessary or clinically indicated, without the necessity of return of the equipment for download. We speculate that ODM may therefore be able to overcome the limitation of the night-to-night AHI variability observed in PSG (27), which is frequently overlooked in clinical practice due to the limitations imposed by the current diagnostic methods for OSA. Moreover, ODM is a simple and reliable method that may be particularly useful for monitoring patients under a variety of treatment modalities, such as the mandibular advancement device, oropharyngeal exercises, position therapy, and weight loss. Patients using CPAP with sub-optimal compliance may also be aided by ODM studies with and without CPAP (28).

Our study has several limitations. First, the study was conducted in a sleep laboratory, and the performance of ODM could possibly decrease in an uncontrolled environment. The number of technical failures related to battery and communication failure were relatively low (6.6%) and within the same range as those observed in HST (∼8%) (29). Moreover, the technical problems were relatively simple (battery charging malfunction) and can be easily solved. Second, the typical HST uses the total recording time to obtain the respiratory disturbance index, whereas PSG-AHI is derived from the sleep time. Therefore, the difference between the methods is directly influenced by sleep efficiency. The current ODM has a built-in accelerometer that can exclude periods of intense movement. However, future studies are necessary to validate an algorithm to help estimate sleep efficiency. Third, the analysis was performed by a proprietary algorithm, which does not allow review and editing of the data. However, PSG is dependent on human analysis and therefore introduces inter-scorer variability (30), which is absent from automatic algorithms. Finally, ODM cannot distinguish between central and obstructive events, and was not used among patients with significant comorbidities, such as heart failure and severe pulmonary diseases.

## CONCLUSION

In conclusion, the performance characteristics of ODM were comparable to the simultaneously performed diagnostic PSG with a lower burden on the participants, and ability to collect and review data on multiple nights at a highly favorable cost. Additional validation in the home setting is needed to confirm the utility of this device as a diagnostic and management tool for OSA.

## CONFLICT OF INTEREST

Biologix provided support in the form of salaries for authors: Andrea Fonseca Cruz, Diego Munduruca Domingues, Pedro Rodrigues Genta Geraldo Lorenzi-Filho is a co-founder of Biologix.

## AUTHOR CONTRIBUTIONS

Each author had full access to the data and takes responsibility for the integrity and accuracy of the analysis. All authors edited the manuscript for important intellectual content and approved the final draft. Role of sponsors: The sponsor had no role in the design of the study, collection and analysis of the data, or preparation of the manuscript.

## Figures and Tables

**Table 1:** Characteristics of the sample population.

		Range
Male (%)	169 (55.6)	
Age, y	55.3±13.8	18-90
BMI, kg/m^2^	30.9±5.7	15.0-52.2
Epworth Sleepiness Scale	11±6	0-24
Comorbidities		
Arterial hypertension (%)	151 (49.7)	
Dyslipidemia (%)	87 (28.6)	
Diabetes mellitus (%)	64 (21.1)	
Depression (%)	26 (8.6)	
Coronary artery disease (%)	20 (6.6)	
Asthma/COPD (%)	15 (4.9)	
PSG		
TRT, min	453.3±36.7	341.0-543.0
TST, min	359.5±62.5	166.0-499.0
SL, min	18.1±24.0	0.0-188.0
WASO, min	75.1±49.9	5.0-285.0
SE, %	79.6±12.6	40.0-98.0
AHI_3%_, events/h	35.3±30.1	0.0-139.7
Moderate-to-severe AHI_3%_ (%)	203 (66.8)	
PSG-ODI_3%_, events/h	32.4±28.2	0.3-129.3
AHI_4%_, events/h	25.6±27.7	0.0-133.6
Moderate-to-severe AHI_4%_ (%)	151 (49.7)	
PSG-ODI_4%_, events/h	23.2±25.5	0.0-119.1
ODM		
TRT, min	470.7±45.4	241.6-662.8
Valid time, min	462.6±46.2	238.5-548.3
ODI_3%_, events/h	30.3±25.9	0.3-130.7
ODI_4%_, events/h	22.8±24.1	0.0-124.9

Data are presented as mean+SD or number and percentage (%).

Abbreviations: BMI=body mass index; OSA=obstructive sleep apnea; COPD=chronic obstructive pulmonary disease; PSG=polysomnography; TRT=total recording time; TST=total sleep time; WASO=wake after sleep onset; SL=sleep latency; SE=sleep efficiency; ODI=oxygen desaturation index; AHI=apnea-hypopnea index; SpO2=oxygen saturation; ODM=overnight digital monitoring.

**Table 2:** Four-class confusion matrix showing classification agreement between the evaluated pairs: A) PSG-AHI_3%_ *vs* PSG-AHI_4%_, B) PSG-AHI_3%_ *vs* ODM-ODI_3%_ and C) PSG-AHI_4%_ *vs* ODM-ODI_4%_.

A)			PSG-AHI3%			
			AHI<5	5≤AHI<15	15≤AHI<30	AHI≥30
	PSG-AHI4%	AHI<5	31	45	5	0
		5≤AHI<15	0	25	42	5
		15≤AHI<30	0	0	23	27
		AHI≥30	0	0	0	101
B)			PSG-AHI3%			
	ODM-ODI3%		AHI<5	5≤AHI<15	15≤AHI<30	AHI≥30
		ODI<4	18	7	0	0
		4≤ODI<12	13	43	7	3
		12≤ODI<25	0	19	46	6
		ODI≥25	0	1	17	124
C)			PSG-AHI4%			
	OD-ODI4%		AHI<5	5≤AHI<15	15≤AHI<30	AHI≥30
		ODI<5	68	9	1	0
		5≤ODI<14	13	46	11	0
		14≤ODI<26	0	16	31	9
		ODI≥26	0	1	7	92

Abbreviations: PSG=polysomnography; AHI=apnea-hypopnea index; ODM=overnight digital monitoring; ODI=oxygen desaturation index.

**Table 3:** Diagnostic performance using the best cutoff for the evaluated pairs: PSG-AHI3% *vs* ODM-ODI3%, and PSG-AHI4% *vs* ODM-ODI4%. PSG AHI3% *vs* PSG AHI4% were compared using the same cutoff, as recommended by the American Academy of Sleep Medicine.

	Cutoffs		
	PSG-AHI4% ≥15 events/h	ODM-ODI3% ≥12 events/h	ODM-ODI4% ≥14 events/h
Sensitivity	74.4%	95.1%	92.1%
Specificity	100.0%	80.2%	88.9%
Accuracy	82.9%	90.1%	90.5%
PPV	100.0%	90.6%	89.1%
NPV	66.0%	89.0%	91.9%
LR+	Infinite	9.7	8.2
LR-	0.5	0.1	0.1

Abbreviations: PSG=polysomnography; AHI=apnea-hypopnea index; ODM=overnight digital monitoring; ODI=oxygen desaturation index; PPV=positive predictive value; NPV=negative predictive value; LR+=positive likelihood ratio; LR-=negative likelihood ratio.
